# First Report of Group CTX-M-9 Extended Spectrum Beta-Lactamases in *Escherichia coli* Isolates from Pediatric Patients in Mexico

**DOI:** 10.1371/journal.pone.0168608

**Published:** 2016-12-19

**Authors:** Jocelin Merida-Vieyra, Agustin De Colsa, Yair Calderon Castañeda, Patricia Arzate Barbosa, Alejandra Aquino Andrade

**Affiliations:** 1 Molecular Microbiology Laboratory, Instituto Nacional de Pediatría [INP], Mexico City, Mexico; 2 Microbiology Department, Escuela Nacional de Ciencias BioloÂgicas, Instituto PoliteÂcnico Nacional, Mexico City, Mexico; 3 Pediatric Infectious Diseases Department, INP, Mexico City, Mexico; 4 Clinical Bacteriology Laboratory, INP, Mexico City, Mexico; Institut Pasteur, FRANCE

## Abstract

The aim of this study was to identify the presence of group CTX-M-9 extended spectrum beta-lactamases (ESBL) in clinical *Escherichia coli* isolates from pediatric patients. A total of 404 non-repeated positive ESBL *E*. *coli* isolates were collected from documented clinical infections in pediatric patients over a 2-year period. The identification and susceptibility profiles were determined using an automated system. Isolates that suggested ESBL production based on their resistance profiles to third and fourth generation cephalosporin and monobactam were selected. ESBL production was phenotypically confirmed using a diffusion method with cefotaxime and ceftazidime discs alone and in combination with clavulanic acid. *bla*_ESBL_ gene identification was performed through PCR amplification and sequencing. Pulsed Field Gel Electrophoresis (PFGE) and Multilocus Sequence Typing (MLST) were performed to establish the clonal relationships of the *E*. *coli* isolates. CTX-M-9-type ESBLs were detected in 2.5% of the isolates. The subtypes corresponded to *bla*_CTX-M-14_ (n = 4) and *bla*_CTX-M-27_ (n = 6). Additionally, coexistence with other beta-lactamases was observed. A clonal relationship was established in three isolates; the rest were classified as non-related. We found seven different sequence type (ST) in CTX-M-9- producing *E*. *coli* isolates. ST38 was the most frequent. This study is the first report in Mexico to document the presence of group CTX-M-9 ESBLs in *E*. *coli* isolates from pediatric patients.

## Introduction

Nosocomial infections constitute one the most important problems of medical care, which do not only increase morbidity-mortality indices, but also the total cost of healthcare. Hospitalized patients in the intensive care units (ICU) are usually more susceptible to these kinds of infections. A prolonged hospital stay, invasive procedures and total parenteral nutrition are some of the principal risk factors for the development of nosocomial infections in pediatric patients [1].

The prevalence of nosocomial infections in pediatric intensive care units (PICUs) has been reported to be 6–12% and 10–25% in neonatal intensive care units (NICUs) [2]. The Enterobacteriaceae are one of the most frequent group of bacteria causing these infections. The selection pressure exerted by the overuse of antibiotics has produced the emergency of multi-drug resistant strains (MDR). Presently, the treatment options for the infections caused by MDR Enterobacteriaceae are limited [3].

*Escherichia coli* is one of the main pathogens that cause infections in both the hospital and community settings and constitutes the principal Enterobacteriaceae in pediatric patients. These infections include urinary tract infections, bacteremia, neonatal meningitis, gastroenteritis, pneumonia, and wound infections [4–6]. Beta-lactams are the most commonly used antibiotics in clinical settings for the treatment of various infections because of their bactericidal action, low toxicity and diverse spectrum. An increase in the resistance to extended-spectrum cephalosporins (ceftriaxone, cefotaxime and ceftazidime) has been observed in *E*. *coli* over the last two decades. This resistance is primarily due to the production of ESBL [7,8]. Among the different ESBL families, CTX-M has become the most prevalent worldwide [9]; both in nosocomial and community acquired infections, principally in urinary tract infections (UTI) [10]. To date, 172 allelic variants have been described for this enzyme [11]. This family is classified into six phylogenetic groups (CTX-M-1, CTX-M-2, CTX-M-8, CTX-M-9, CTX-M-25 and KLUC) according to amino acid sequence similarities [10]. CTX-M-1 and CTX-M-9 are the most frequently described groups, with CTX-M-15 the most common subtype worldwide, followed by CTX-M-14, CTX-M-2, CTX-M-3 and CTX-M-1 [12].

In an epidemiological surveillance report on antimicrobial susceptibility profile conducted in 11 Latin America countries in 2011, a prevalence of ESBL-Producing *E*. *coli* was 7–71% with an average of 37%. In Mexico, the prevalence was 71% [13]. In another surveillance study carried out by SENTRY program from 2011–2014 in 21 hospital centers, ESBL-producing *E*. *coli* prevalence was found to vary from 14.7 to 69.9%. In Mexico, the prevalence reported was 69.9% [14].

In 2014, a total of 5771 Enterobacteriaceae isolates were collected in 69 hospital centers in US as a part of the International Network for Optimal Resistance Monitoring (INFORM) program, 13.6% presented ESBL phenotype and of these,14.5% was *E*. *coli*. The most common enzyme was CTX-M-15 (49.9%) followed by CTX-M-14 (20.5%) [15].

Another study by the SENTRY program, carried out in nine European countries in 2009–2011, evaluated 2544 Enterobacteriaceae isolates from ICU. 709 of this total were *E*. *coli* and ESBL phenotype was reported in 16.6% [16].

In Spain and some regions of Asia, CTX-M-9 is the predominant group [6,17]. In China, the presence of CTX-M-Producing *E*. *coli*, mainly the group CTX-M-9, in urinary tract infections was documented [18].

In South America, the group CTX-M has been found to reach endemic proportion [19]. In Colombia, there are reports of CTX-M-1 [20] while in Ecuador, CTX-M-1, CTX-M-2 and CTX-M-9 have been documented [21]. Group CTX-M-9 also has been described in Bolivia (43%), Argentina (6%) and Brazil (1.2%) [22–24]. In Mexico, several studies have reported the presence of group CTX-M-1 (subtype CTX-M-15) [25–29]. However, no studies have documented the molecular and epidemiological characteristics of CTX-M-9 ESBL-producing *E*. *coli* isolates in the pediatric population. The aim of this study was to describe the presence of group CTX-M-9 ESBL in *E*. *coli* isolates obtained from pediatric patients.

## Materials and Methods

### Isolate collection

During a two-year period (February 2013—January 2015), a total of 404 non-repeated *E*. *coli* isolates with ESBL phenotype were collected at the Instituto Nacional de Pediatria (INP), that is a tertiary care hospital in Mexico City. These isolates were obtained from various clinical samples causing infections in pediatric patients (0–18 years of age) and were identified using the Phoenix^®^ Automated Microbiology System (Becton Dickinson, USA).

### Ethics statement

The study was approved by INP Ethic Committee (reference number 066/2013). The Review Board exempted request for informed consent because the bacteria included in the study were obtained by routine procedures and did not affect the patients.

### Susceptibility tests

The susceptibility profiles of the isolates were determined using a Phoenix^®^ Automated Microbiology System (Becton Dickinson, USA). The results were interpreted according to the guidelines of the Clinical and Laboratory Standards Institute (CLSI, 2015) [30]. The isolates were selected based on their resistance profiles to third generation (ceftriaxone and ceftazidime) and fourth generation (cefepime) cephalosporins and monobactams (aztreonam).

### ESBL confirmatory tests

The phenotypic detection of ESBL was performed by the combined disc method using sensi-discs of cefotaxime and ceftazidime alone or in combination with clavulanic acid according to the CLSI guidelines [30].

The presence of ESBL was confirmed by an increase of ≥5 mm in the diameter of the zone of inhibition for any agent tested in combination with clavulanic acid compared with the diameter of the zone of inhibition for the agent alone. *Klebsiella pneumoniae* ATCC 700603 and *E*. *coli* ATCC 25922 were used as the positive and negative controls, respectively.

### Molecular characterization of the genes encoding ESBL

DNA extraction was performed using a QIAamp® DNA Mini kit (QIAGEN, Hilden, Germany) following the manufacturer’s instructions. The molecular detection of beta-lactamases (*bla*_CTX-M-1_, *bla*_CTX-M-2_, *bla*_CTX-M-9_, *bla*_CTX-M8/25_, *bla*_TEM_ and *bla*_SHV_) was performed by end-point PCR using previously reported primers [31,32]. The reaction was performed in a GenAmp ® PCR System 9700 (Applied Biosystems). The final volume of the reaction mixture was 25 μL and contained 12.5 μL of AmpliTaq Gold® 360 MasterMix (Applied Biosystems, Foster City, CA, USA) and 0.4 pmol/μL of the primers. The obtained fragments were purified and sequenced in an ABI Prism 310 analyzer. The sequences were analyzed with nBLAST program [33,34], and analyzed using multiple alignments with the BioEdit program v7.2.5.0 (Ibis Biosciences, Carlsbad, CA, USA) to determine the beta-lactamase subtype.

### Molecular typing

The clonal relationship of the isolates was determined using the pulse-field gel electrophoresis (PFGE) technique in a CHEF Mapper XA System (Bio-Rad Laboratories, Hercules, CA, USA) after digestion of the chromosomal DNA with 50 U of the XbaI restriction enzyme (Invitrogen, Carlsbad, CA, USA). The technique was performed by following the PulseNet *E*. *coli* O157:H7 protocol (CDC, Atlanta, GA, USA) [35]. *Salmonella enterica* serovar Braenderup ATCC BAA-664 was used as the molecular size marker. The gel was stained with ethidium bromide (10 mg/mL) and visualized with UV light in a gel imaging system. The patterns were interpreted using the Tenover criteria [36].

MLST analysis was performed by amplifying seven housekeeping genes (*adk*, *fumC*, *gyrB*, *icd*, *mdh*, *purA* and *recA)* with primers and conditions previously described [37]. The sequences were analyzed in the program and database available in the site http://mlst.warwick.ac.uk/mlst/.

## Results

The 404 *E*. *coli* isolates with ESBL patterns were positive for the phenotypic confirmatory test. Group CTX-M-9 ESBLs were detected in 2.5% of these isolates (n = 10). The resistance profile to beta-lactam of the isolates with CTX-M-9 is shown in Table 1.

**Table 1:** Resistance profile and Molecular Characteristics of the CTX-M-9-Producing *E*. *coli* Isolates.

Isolate	CRO	CAZ	FEP	AZT	PTZ	IMP	MEM	ERT	ESBL	beta-lactamases genes				Typing	
	MIC (μg/mL)									TEM	SHV	CTX-M-1	CTX-M-9	PFGE	MLST
**EC001**	≥64	64	>16	>16	4/4	≤1	≤0.25	≤0.5	+	1*	-	15	14	NR	ST942
**EC230**	≥64	8	>16	16	64/4	≤1	≤0.25	≤0.5	+	1*	-	79	27	NR	ST457
**EC284**	16	16	16	4	≤2/4	≤1	≤0.25	≤0.5	+	1*	-	-	27	NR	ST354
**EC295**	≥64	4	>16	8	4/4	≤1	≤0.25	≤0.5	+	-	-	-	14	NR	ST38
**EC300**	≥64	256	>16	>16	>64/4	≤1	≤0.25	≤0.5	+	-	-	15	14	NR	ST5489
**EC326**	16	8	8	8	≤2/4	≤1	≤0.25	≤0.5	+	-	-	-	27	NR	ST69
**EC419**	≥64	8	>16	16	≤2/4	≤1	≤0.25	≤0.5	+	-	-	-	27	A2	ST38
**EC579**	≥64	8	8	8	≤2/4	≤1	≤0.25	≤0.5	+	-	-	-	27	A	ST38
**EC600**	16	8	>16	16	≤2/4	≤1	≤0.25	≤0.5	+	-	-	-	27	A	ST38
**EC719**	>32	2	16	2	≤2/4	≤1	≤0.25	≤0.5	+	1*	-	-	14	NR	ST648

CRO: ceftriaxone, CAZ: ceftazidime, FEP: cefepime, AZT: aztreonam, PTZ: piperacillin/tazobactam IMP: imipenem, MEM: meropenem, ERT: ertapenem, ESBL: extended spectrum beta-lactamases, NR: Non-Related. PFGE: Pulsed Field Gel Electrophoresis, MLST: Multilocus sequence

* non ESBL.

The multiple sequence alignment revealed that CTX-M-14 (n = 4) and CTX-M-27 (n = 6) were the allelic variants found. Additionally, coexistence with other types of beta-lactamases was observed in five isolates (TEM-1, CTX-M-15 and CTX-M-79) (Table 1).

**Fig 1:**
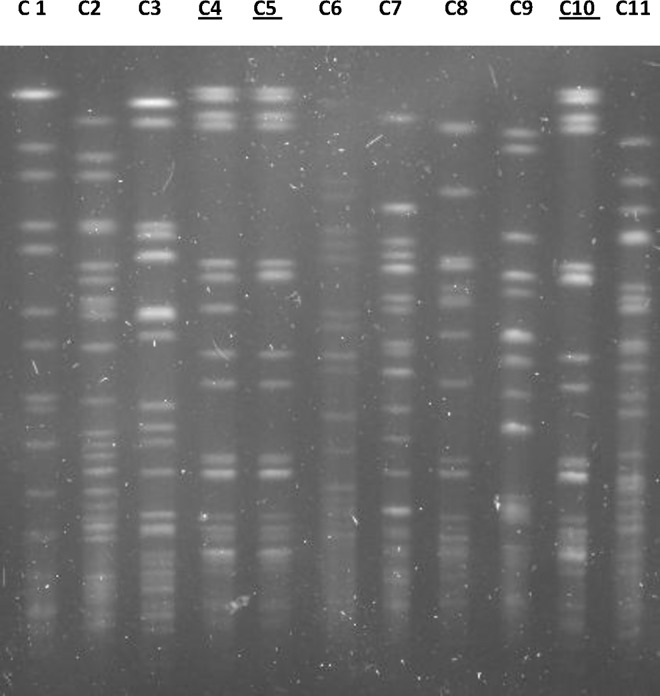
PFGE of the CTX-M-9-Producing *E*. *coli* Isolates. C1: *Salmonella enterica* serovar Braenderup ATCC BAA-664, C2: EC230, C3: EC326, C4: EC419, C5: EC579, C6: EC01, C7: EC284, C8: EC295, C9: EC300, C10: EC600, C11: EC719. The clonally related isolates are underlined: EC579 and EC600 (clone A) and EC419 (clone A2).

Regarding the molecular typing, we found that two isolates (EC579 and EC600) belonged to the same clone and were closely related to EC419. The rest of the isolates were classified as non-related (Fig 1). The isolates were obtained from different clinical samples (Table 2).

**Table 2:** Epidemiological and Clinical Characteristics of the Patients with CTX-M-9-Producing *E*. *coli* Isolates.

Isolate	Gender	Age	Date	Service	Diagnosis	Infection	Sample	Treatment		Otucome
								Previous*	Definitive	
**EC001**	M	17	02/05/13	ID	ALL(relapse)	Septic shock	Blood culture	Cefepime, ceftriaxone, meropenem	Meropenem	Death
**EC230**	F**	4	11/06/13	Nephrology	CNS,CKD	Odontegenic abscess	Abscess aspirate	Cefepime, ceftriaxone, cefuroxime	Abscess drainage	Alive
**EC284**	M	14	01/22/14	Hematology	AML-M6	Septic shock, neutropenic, colitis	Blood culture	Ceftriaxone, Pip-Tazob, meropenem	Meropenem	Alive
**EC295**	M	3	01/30/14	Oncology	Histiocytosis	Perianal cellulitis-abscecs	Secretion	Ceftriaxone, cefepime, meropenem	Cefepime, abscess drainage	Alive
**EC300**	M	3	01/30/14	ID	Lennox-Gastaut Syndrome, malnutrition	Pneumonia	BAL	Ceftriaxone, cefepime	Meropenem	Alive
**EC326**	F**	4	02/21/14	Nephrology	CNS,CKD	Hemodialysis, cateter side infection	Wound secretion	Cefepime, ceftriaxone, cefuroxime	Ceftriaxone, wound care	Alive
**EC419**	F	1	06/13/14	ID	VUR grade IV, dysmorphic syndrome	UTI (recurrent)	Urine	Ceftriaxone, ertapenem (4 courses in 8 months)	Ertapenem	Alive
**EC579**	F	7	10/27/14	Surgery	Complicated appendecitis	Surgical site infection	Wound secretion	Ceftriaxone	Ciprofloxacin, wound care	Alive
**EC600**	F	10	11/03/14	Nephrology	CKD	Peritonitis	Peritoneal fluid	None	Ertapenem	Alive
**EC719**	F	2	01/23/15	HSCTU	Post-HSCT, AML-M5	CRBSI	Blood culture	Cefepime	Meropenem	Death***

*ID: Infectious Diseases, Treatment with *beta-lactam antibiotics* in the previous three months

** Same patient

*** Not related to *E*. *coli* infection, HSCTU: Hematopoietic Stem Cell Transplant Unit; ALL: Acute Lymphoblastic Leukemia; AML: Acute Myeloid Leukemia; CNS: Congenital Nephrotic Syndrome; CKD: Chronic Kidney Disease; VUR: Vesico-Ureteral Reflux; UTI: Urinary Tract Infection; CRBSI: Catheter-Related Blood Stream Infection; BAL: Bronchoalveolar Lavage, Pip-Tazob: piperacillin/tazobactam.

Seven different type sequences (ST) were found in ten CTX-M-9-producing *E*. *coli* isolates (Table 1). The most frequent was ST38 (n = 4). In three, the subtype CTX-M-27 was detected and CTX-M-14 in one. The three isolates with CTX-M-27 (EC419, EC579 and EC 600) were clonally related (Fig 1), while EC295 isolate did not show similarity in the profile obtained with PFGE. Two isolates were from the same patient; however, both have different ST (EC230-ST457 and EC326-ST69).

For the clinical and epidemiological analysis, we studied the relationship between the patients and the *E*. *coli* CTX-M-9. All infections were nosocomial. Eight of the ten patients had a chronic underlying pathology requiring previous hospitalization, whereas two patients were newly admitted to the hospital (EC600 and EC579) (Table 2). All the patients with the exception of one had received third generation cephalosporins in the previous three months, while four of them had also previously received carbapenems.

A total of 78% of the patients had some degree of immunosuppression. The main isolation sites were blood (30%) and secretions derived from skin and soft tissue infections (30%) (Table 2). The first isolate was reported in February 2013. In 2014, the highest number of isolates was reported (n = 7). In January 2014, three patients coincided temporally (EC295, EC300, and EC326) but not in clinical services or enzymatic patterns.

Three cases were reported in the nephrology service, with two in the same patient at a three-month interval. The two infections were caused by a different enzyme subtype and by non-clonally related isolates: the first infection was caused by *E*. *coli* with a combination of CTX-M-79 and CTX-M-27 (EC230) and the second was caused by *E*. *coli* bearing CTX-M-27 (EC326). The other nephrology patient arrived almost seven months later and had a non-related CTX-M-14-producing isolate.

The two patients were infected after being hospitalized for the first time (EC579 and EC600). Both patients were admitted through the pre-hospitalization service in a two-week interval. These two isolates belonged to clone A, with subtype CTX-M-27, both corresponding ST38. Isolate EC419 was closely related to clone A (A2). However, there was no temporal or clinical service relationship between the isolates with this pattern.

In this series, six patients received definitive treatment with carbapenems and one of them with ciprofloxacin. In three of the infections, although carbapenems was not administered, the infection was resolved draining the abscess and local management of the wound (Table 2). Two of the patients died; however, one of them, death was not related with the *E*. *coli* CTX-M-9 infection. An important aspect to note is that one patient suffered two *E*. *coli* CTX-M-9 infections in two different times in a period of three months; however, *E*. *coli* isolates (ST457 and ST69) were not related (Table 1).

## Discussion

This study represents the first finding of group CTX-M-9 ESBLs in *E*. *coli* isolates from a pediatric population in Mexico. These enzymes were found in 2.5% of the isolates. The subtypes corresponded to CTX-M-14 and CTX-M-27. The CTX-M-1 group has been previously described in our country in several studies; the most frequent subtype found was CTX-M-15, reaching up 85% of *E coli* isolates with ESBL phenotype [25–29]. Although CTX-M-15 is an ESBL with a worldwide distribution, group CTX-M-9 has been reported to be the primary group in some regions of Europe and Asia. In Japan, 127 out of 165 *E*. *coli* ESBL isolates (77%) belonged to group CTX-M-9 [38]. In Spain, 96 cases of nosocomial bacteremia by ESBL-producing *E*. *coli* were reported in a study conducted in 13 hospitals. CTX-M-14 was present in 48% of these isolates, followed by CTX-M-27 (14%) [39]. In Korea, CTX-M-14 was found in 39% (n = 32) and CTX-M-15 in 33% (n = 27) of 82 ESBL-producing *E*. *coli*, whereas CTX-M-27 was found in only one isolate [40]. In China, 77 out of 197 ESBL-producing *E*. *coli* isolates were CTX-M-14 (39%), followed by CTX-M-15 with 64 isolates (32%) [41].These findings indicate that CTX-M-14 and CTX-M-15 have become the predominant subtypes worldwide [42].

The presence of enzymes from group CTX-M-9 has also been reported in healthy individuals colonized with *E*. *coli*. In Spain, the prevalence of ESBL-producing *E*. *coli* was 5.06% in a study performed with colonized individuals, of which group CTX-M-9 was the most frequent (54%); CTX-M-14 (n = 74, 91.35%) was the main subtype of this group of enzymes [43]. In Bolivia, 43% of 60 *E*. *coli* ESBL isolates from healthy children belonged to group CTX-M-9 [24].The presence of CTX-M-14 and CTX-M-27 has been reported in water, fish and fresh vegetables. These studies show that the community and the environment are also important reservoirs of CTX-M-9 enzyme producers. The identification of these reservoirs is important to identify possible transmission pathways for the acquisition of *E*. *coli* isolates producing this group of enzymes [44,45].

Coexistence with other ESBLs was only observed in five of the ten isolates producing group CTX-M-9 ESBLs. These examples corresponded to enzymes from group CTX-M-1 (CTX-M-15 and CTX-M-79). TEM-1 beta-lactamase was also found, although they were not extended spectrum. The coexistence of CTX-M-14 and SHV-12 has been reported in Korea [40], and the coexistence of CTX-M-14 and CTX-M-15 has been reported in the U.S. [46]. The dissemination of *bla*_CTX-M-27_ typically occurs through plasmids of the IncF incompatibility group[47], whereas *bla*_CTX-M-14_ is found in the IncK and IncH12 plasmids [48,49], *bla*_CTX-M-14_ has been reported to be located in the chromosome [50]. CTX-M-27 can be differentiated from CTX-M-14 by the substitution of an amino acid, D240G. This change confers a higher hydrolytic activity against ceftazidime [51].

The dissemination of *E*. *coli* isolates producing CTX-M-9 is usually non-clonal. The association of CTX-M-14 with different sequence types has been reported [52, 53]. However, there are reports of some outbreaks produced by only one *E*. *coli* clone. In Spain, an outbreak in 2011 caused by a CTX-M-14-producing *E*. *coli* occurred in a neonatal intensive care unit. Out of 25 isolates producing CTX-M-14, 21 showed the same clonal pattern; the remaining four were classified as non-related. Using MLST, these *E*. *coli* isolates were shown to belong to clone ST23 [54]. The most frequent ST in CTX-M-9 producing isolates was ST38 (n = 4). Countries as China, Japan, Korea, Sweden, Netherlands and United Kingdom have been reported *E*. *coli* ST38 with the subtype CTX-M-14 in human infections [50, 55–59]. In Korea, ST38 was reported as the second most frequent ST in CTX-M-14 producing *E*. *coli* isolates of UTI. The rate of incidence of this ST reached 27.5% [59]. Also, it has been obtained from other sources like healthy human, water and pets [60–62].

*E*. *coli* ST648 associated to the CTX-M-14 enzyme, as seen in EC719 isolate, has been isolated in humans and other sources like water [58–60]. ST648 with CTX-M-9 has been isolated from animals like dogs, cats and birds [62–63].

*E*. *coli* ST354 and ST69 (EC230 and EC326, respectively) have also been detected in Korea and Hong Kong in *E*. *coli* with CTX-M-14 [58,59]. In our study, these ST were found associated to CTX-M-27.

In other regions of the world, some ST that were found in this analysis, have been associated with the presence of ESBL, non-ESBL and carbapenemases. In China, clinical isolates of *E*. *coli* ST38, ST648 and ST69 with type 2 *Klebsiella pneumoniae* carbapenemase (KPC-2) and CTX-M-14 were described [64]. Likewise, *E*. *coli* ST69 with CTX-M-15 has been found in animals [62].

In Japan, the emergency of New Delhi metalo-beta-lactamase (NDM-1)-producing *E*. *coli* ST38 was reported in an isolate from blood culture [65]. In Korea, a patient was colonized with *E*. *coli* ST457 harboring carbapenemase OXA-232 [66]. In Australia and China, ST648 has been associated with the presence of CTX-M-1 and CTX-M-15 [60, 61]. In US, ST648 isolates from pets with CTX-M-15, CTX-M-1 and SHV-12 have been found [62]. In Australia, ST354 with CTX-M-9, CTX-M-1 and CTX-M-15; and ST457 with CTX-M-15 and CMY were isolated from dog feces [61]. These descriptions show that these ST are associated with the dispersion of other resistance mechanisms that does not only involve ESBL but other enzymes, like the carbapenemases; situtation that can seriously complicate the treatment of *E*. *coli* infections.

The association of CTX-M-14 and CTX-M-27 with the pandemic clone ST131 has been reported [67]. Recently, the presence of *E*. *coli* ST131 with CTX-M-27 and *E*. *coli* ST226 with CTX-M-14 in humans has been described in Portugal [68].

We found four isolates with different ST with non-ESBL TEM-1 and three isolate with enzymes of the group CTX-M-1. In two of them, we found more than one beta-lactamase: EC01 (TEM-1 and CTX-M-15) and EC230 (TEM-1 and CTX-M-79).

This report is not intended to focus on the risk factors for acquiring *E*. *coli* with ESBL; however, in this series we found that eight out of ten patients had a chronic underlying pathology with long hospital stay or frequent admissions and discharges; thus, these patients were probably colonized previously. All except one patient, had previously received third generation cephalosporins. Indeed, the factors such as: previous use of antibiotics, prolonged hospital stay, neurological diseases and urinary tract anomalies among others, are associated with the acquisition of ESBL-producing Enterobacteriaceae [3,4]. Our results suggest the endogenous circulation of *E*. *coli* with these enzymatic patterns in our hospital. It is of the outmost to have an active surveillance program for timely detection of both infected and colonized patients by Enterobacteriaceae harboring emerging resistance mechanisms. Such program would optimize the prevention, control and management of these infections.

## Conclusions

This is the first report in Mexico of *E*. *coli* isolates obtained from pediatric patients producing group CTX-M-9 ESBLs (CTX-M-14 and CTX-M-27). The most frequent ST was ST38, which has been associated with the production of this type of enzymes in other countries. All of the infections were of nosocomial origin, but none of them were part of an outbreak. It is necessary to continue epidemiological and molecular surveillance to improve the prevention and control of infections associated with healthcare.
